# Cystatin F as a neuroimmune effector in CNS inflammation and neurodegeneration

**DOI:** 10.3389/fcell.2026.1959727

**Published:** 2026-09-22

**Authors:** Kaitlin Murray, Kellie Fernandez, Thomas E. Lane

**Affiliations:** 1 Department of Neurobiology and Behavior, School of Biological Sciences, University of California, Irvine, CA, United States; 2 Department of Molecular Biology and Biochemistry, School of Biological Sciences, University of California, Irvine, CA, United States; 3 Center for Virus Research, University of California, Irvine, CA, United States

**Keywords:** cystatin F, inflammation, neurodegeneration, neuroimmunology, protease inhibitor

## Abstract

Cystatin F (*CST7*) has emerged as one of the most consistently upregulated genes identified in transcriptomic and spatial profiling studies of the central nervous system (CNS), particularly within activated microglia and other neuroimmune cell populations. Once regarded primarily as a marker of immune activation, accumulating evidence suggests that the encoded protein, cystatin F, may play a more active role in regulating neuroimmune function through its established ability to modulate lysosomal cysteine proteases. This review synthesizes current knowledge of cystatin F biology and examines its growing relevance across aging, neurodegenerative disease, and CNS inflammation. We first summarize the molecular biology of cystatin F, including its unique intracellular processing, activation, and regulation of cathepsin activity. We then discuss evidence supporting *CST7* as a conserved marker of activated neuroimmune cells across diverse immune populations and disease-associated cellular states. Next, we review findings implicating *CST7* across neurodegenerative diseases, highlighting remarkable consistency across distinct pathological contexts. Finally, we examine potential mechanisms linking cystatin F to lysosomal homeostasis, antigen presentation, inflammatory signaling, and cell death pathways. Although direct functional studies within the CNS remain limited, the convergence of biochemical, immunological, and transcriptomic evidence suggests that cystatin F is well positioned to influence neuroimmune responses rather than merely reflect them. Defining the precise role of cystatin F in CNS immunity may therefore provide new insight into the regulation of neuroinflammation and identify novel opportunities for therapeutic intervention across a broad spectrum of neurological diseases.

## 1 Introduction

Neuroinflammation is now recognized as a central feature of numerous neurological disorders, including neurodegenerative diseases, autoimmune disorders, traumatic injury, and central nervous system (CNS) infections (Heneka et al., 2025; Shi and Yong, 2025). Although these conditions differ considerably in their initiating insults, they share a common response characterized by activation of resident microglia, recruitment of peripheral immune cells, and extensive remodeling of the neuroimmune environment (Ransohoff, 2016; Harry and Kraft, 2008; Yang et al., 2025). Rather than representing a passive consequence of tissue injury, these immune responses actively shape disease progression by influencing pathogen clearance, protein aggregation, synaptic remodeling, and neuronal survival. Consequently, understanding the molecular pathways that regulate neuroimmune cell function has become a major focus of contemporary neuroscience.

Over the past decade, advances in single-cell transcriptomic technologies have fundamentally changed our understanding of CNS immunity (Keren-Shaul et al., 2017; Masuda et al., 2019; Hammond et al., 2019). These approaches have revealed remarkable heterogeneity among microglia and other immune populations and identified conserved activation states that recur across diverse neurological diseases. As a result, numerous genes have emerged as reproducible markers of activated neuroimmune cells. However, for many of these genes it remains unclear whether altered expression simply reflects changes in cellular state or whether they actively regulate immune function and disease pathogenesis.

Among these emerging candidates, *CST7*, which encodes the lysosomal cysteine protease inhibitor cystatin F, has become one of the most consistently identified genes associated with activated neuroimmune populations (Hammond et al., 2019; Keren-Shaul et al., 2017; Krasemann et al., 2017; Masuda et al., 2019). Initially characterized as a hematopoietic-restricted gene expressed in cytotoxic lymphocytes, *CST7* has subsequently been identified in activated microglia across aging, neurodegenerative disease, CNS inflammation, and injury (Hamilton et al., 2008; Santamaría et al., 1999; Keren-Shaul et al., 2017; Krasemann et al., 2017). Importantly, unlike many genes identified through large-scale transcriptomic studies as markers of activated cell states, cystatin F possesses a well-defined biochemical function. Through regulated inhibition of lysosomal cysteine proteases, it is positioned to influence biological processes fundamental to immune cell function, including antigen presentation, lysosomal homeostasis, inflammatory signaling, and cytotoxic activity (Hamilton et al., 2008; Colbert et al., 2009; Magister and Kos, 2013). These observations raise an important question: does *CST7* simply mark activated neuroimmune states, or does cystatin F actively contribute to their regulation?

In this review, we examine current knowledge of cystatin F biology and summarize evidence linking *CST7* expression to neurodegenerative disease and CNS inflammation. We first summarize the molecular biology, processing, and protease targets of cystatin F before reviewing *CST7* expression across neuroimmune cell populations and disease contexts. We then discuss potential mechanisms through which cystatin F may regulate lysosomal function, antigen presentation, inflammatory signaling, and cell death. Finally, we consider whether the available evidence supports viewing *CST7* primarily as a biomarker of neuroimmune activation or cystatin F as a functional regulator of CNS immunity. By integrating biochemical, immunological, and transcriptomic evidence, we propose that cystatin F represents an important candidate neuroimmune effector whose functions in the CNS are only beginning to be understood. The recurring induction of *CST7* across major neurodegenerative and neuroinflammatory conditions and the established and proposed mechanisms through which cystatin F may regulate neuroimmune function are summarized (Figure 1).

**FIGURE 1:**
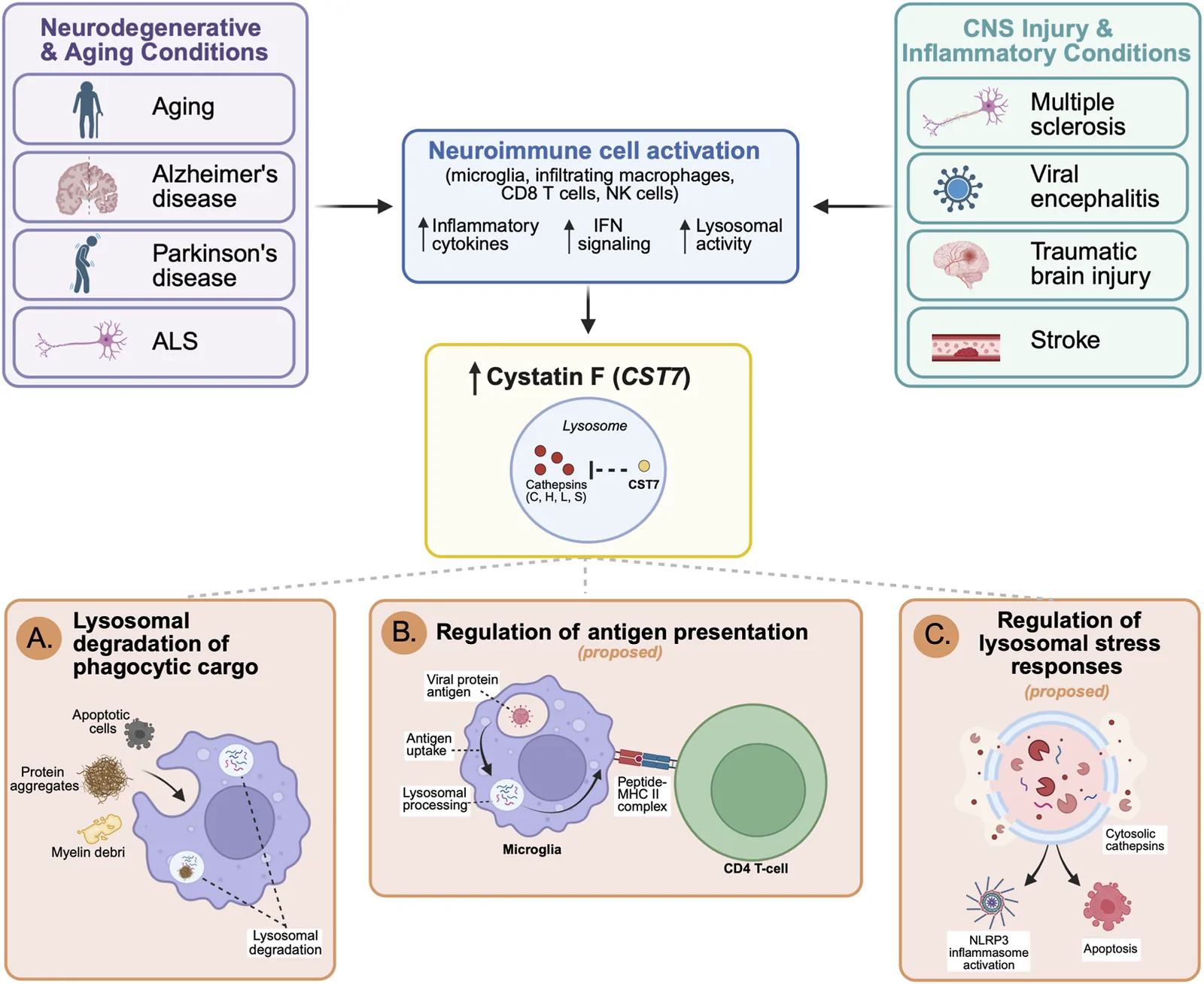
*CST7* induction across neurodegenerative and neuroinflammatory conditions and proposed mechanisms by which cystatin F may regulate neuroimmune function. Neurodegenerative disorders (aging, Alzheimer’s disease, Parkinson’s disease, and amyotrophic lateral sclerosis) and CNS injury/inflammatory conditions (multiple sclerosis, viral encephalitis, traumatic brain injury, and ischemic stroke) are consistently associated with activation of neuroimmune cells, including microglia, infiltrating macrophages, and, in some contexts, cytotoxic lymphocytes. These activated cell populations exhibit enhanced inflammatory signaling, interferon responses, and lysosomal activity, coinciding with increased expression of *CST7*, which encodes the lysosomal cysteine protease inhibitor cystatin F. Current evidence supports a role for cystatin F in regulating lysosomal protease activity and degradative function **(A)**. Based on its established biochemical activity and the known functions of lysosomal cathepsins, cystatin F may also influence antigen processing and MHC class II antigen presentation **(B)** and modulate lysosomal stress responses by limiting excessive cathepsin activity, thereby potentially reducing inflammasome activation and protease-mediated cell death **(C)**. While lysosomal protease regulation is supported by experimental evidence, the roles depicted in panels B and C remain conceptual models that require direct validation in CNS-resident immune cells.

## 2 Biology of cystatin F

Before examining *CST7* expression in neuroimmune cells and neurological disease, it is important to understand the biology of its encoded protein, cystatin F. The unique structural, trafficking, and processing characteristics of cystatin F distinguish it from other members of the cystatin family and underlie its ability to regulate lysosomal protease activity. This section summarizes the key molecular and biochemical features of cystatin F that provide the foundation for the studies discussed in subsequent sections.

### 2.1 The cystatin superfamily and cystatin F at a glance

Cystatin F is a member of the type II cystatin family within the larger cystatin superfamily of cysteine protease inhibitors (Ni et al., 1998). Cystatins are reversible, tight-binding inhibitors of papain-like cysteine proteases, with several family members also capable of inhibiting legumain (Alvarez-Fernandez et al., 1999). Through the regulation of protease activity, cystatins contribute to diverse biological processes including antigen presentation, inflammation, and tissue remodeling (Henskens et al., 1996; Ochieng and Chaudhuri, 2010; Nathanson et al., 2002).

The cystatin superfamily includes type I cystatins, also known as stefins; type II cystatins; type III cystatins, or kininogens; and non-inhibitory type IV cystatin-like proteins. Type I cystatins are predominantly cytosolic, whereas type II cystatins generally contain signal peptides and enter the secretory pathway, although they may also be retained or internalized within cells. Type III cystatins are larger multidomain proteins commonly found in body fluids (Magister et al., 2012). Inhibitory cystatins share three conserved elements: a glycine residue near the N-terminus, a central QXVXG motif in the first hairpin loop, and a PW-containing motif in the second hairpin loop. Together, these regions form a wedge-shaped inhibitory surface that inserts into the active-site cleft of papain-family cysteine cathepsins and blocks substrate access (Turk and Bode, 1991).

Although inhibitory cystatins share this basic architecture, individual family members differ in their cellular distribution, subcellular localization, activation, and protease selectivity. Cystatin F is particularly distinct because its expression is concentrated in hematopoietic cells and because it is synthesized as an inactive disulfide-linked dimer that must undergo intracellular processing to inhibit papain-family cathepsins efficiently (Halfon et al., 1998; Alvarez-Fernandez et al., 1999). Further N-terminal proteolysis alters its target repertoire and enables inhibition of cathepsin C. Cystatin F is also N-glycosylated, allowing secreted protein to be internalized and delivered to the endolysosomal compartments of neighboring cells (Colbert et al., 2009; Nathanson et al., 2002). These properties distinguish cystatin F from more broadly expressed type II family members such as cystatin C and allow its activity to be regulated at multiple levels.

### 2.2 Gene and protein architecture

Type II cystatins contain an N-terminal signal peptide of approximately 19–28 residues that directs translocation into the endoplasmic reticulum during biosynthesis, as well as two conserved intrachain disulfide bonds that stabilize the cystatin fold (Abrahamson et al., 2003). The human *CST7* gene is located within the type II cystatin gene cluster on chromosome 20p11.21–p11.22, whereas the murine ortholog, *Cst7*, is located on chromosome 2 in the G1–G3 region (Ni et al., 1998). Both the human and murine genes contain four exons, and the human gene spans approximately 11 kb (Morita et al., 2000).

Cystatin F shares approximately 27%–33% sequence identity with other type II cystatins but contains several distinctive structural features, including an extended N-terminal region and non-conservative substitutions within its protease-binding surface (Hamilton et al., 2008). In the second hairpin loop, cystatin F contains a tryptophan residue at a position occupied by tyrosine in many other cystatins and also carries a charged residue within the conserved QXVXG motif (Ni et al., 1998). These substitutions contribute to differences in protease recognition and inhibitory activity.

In addition to the two intrachain disulfide bonds characteristic of type II cystatins, cystatin F contains two additional cysteine residues, Cys26 within the extended N-terminus and Cys63 within the cystatin fold. These residues form reciprocal intersubunit disulfide bonds linking Cys26 of one monomer to Cys63 of the other, thereby stabilizing an inactive cystatin F dimer (Schüttelkopf et al., 2006; Hamilton et al., 2008). Dimerization obstructs the papain-family protease-binding surface and provides an additional mechanism through which cystatin F activity is controlled.

### 2.3 Intracellular trafficking, secretion, and uptake

Cystatin F is one of only two N-glycosylated type II cystatins, the other being cystatin E/M. Human cystatin F contains N-linked glycosylation sites at Asn62 and Asn115, whereas murine cystatin F retains only the glycosylation site corresponding to Asn62. Glycosylation at Asn62 is required for intracellular targeting, while the glycan at Asn115 does not appear to carry targeting information (Hamilton et al., 2008).

During transit through the secretory pathway, the high-mannose glycan at Asn62 can acquire a mannose-6-phosphate tag. This modification permits recognition by the cation-independent mannose-6-phosphate receptor and delivery of cystatin F to endolysosomal compartments, where reduction and proteolytic processing generate active forms of the inhibitor (Colbert et al., 2009). However, a portion of mannose-6-phosphate-tagged cystatin F is constitutively secreted rather than retained intracellularly.

Secreted dimeric cystatin F remains largely inactive against papain-family cathepsins but can be internalized by cells expressing the cation-independent mannose-6-phosphate receptor. Following uptake, it is transported to endolysosomes and converted into active forms through disulfide reduction and N-terminal processing (Colbert et al., 2009). Cystatin F can therefore regulate intracellular protease activity both within the cell in which it is produced and, potentially, within neighboring cells. This capacity for intercellular transfer distinguishes cystatin F from conventional cell-autonomous protease inhibitors and introduces an autocrine or paracrine dimension to its function.

### 2.4 Protease targets and activation

Cystatin F is secreted primarily as a dimer held together by two intersubunit disulfide bonds that obstruct the canonical papain-family protease-binding sites (Hamilton et al., 2008). In this dimeric state, cystatin F is inactive against C1 papain-family cathepsins but retains the ability to interact with legumain, an asparaginyl endopeptidase belonging to the C13 cysteine protease family (Alvarez-Fernandez et al., 1999).

To acquire inhibitory activity against papain-family cathepsins, the dimer must undergo disulfide reduction within the endocytic pathway, releasing an active monomeric form (Langerholc et al., 2005; Hamilton et al., 2008; Colbert et al., 2009). Intact monomeric cystatin F inhibits several C1 cysteine cathepsins with differing affinities. Biochemical studies indicate stronger inhibition of cathepsins V, F, L, and K than of cathepsins S and H, with little or no inhibition of cathepsins C, X, and B by the intact monomer (Langerholc et al., 2005; Hamilton et al., 2008; Perišić Nanut et al., 2017).

The protease selectivity of cystatin F changes following removal of its extended N-terminus. N-terminally processed cystatin F gains the ability to inhibit cathepsin C, whose partially occluded active site is accessible only to inhibitors with sufficiently short N-terminal regions (Hamilton et al., 2008). This processing-dependent change is particularly important in cytotoxic immune cells, in which cathepsin C participates in the activation of granule-associated serine proteases.

Cystatin F inhibits legumain through a separate binding surface located on the opposite face of the protein from the papain-family inhibitory site. Because this surface remains accessible in both the monomeric and dimeric forms, cystatin F can inhibit legumain without first undergoing monomerization (Alvarez-Fernandez et al., 1999; Schüttelkopf et al., 2006). Thus, dimer reduction and N-terminal proteolysis do not simply activate cystatin F but generate molecular forms with distinct protease specificities.

### 2.5 Expression and immune-cell functions

Unlike broadly expressed type II cystatins such as cystatin C, cystatin F is expressed predominantly by immune cells (Halfon et al., 1998). High expression has been reported in T cells, natural killer cells, monocytes, and dendritic cells (Halfon et al., 1998; Ni et al., 1998; Perišić Nanut et al., 2017). Its expression pattern is consistent with the prominent roles of cysteine cathepsins in immune-cell differentiation, antigen processing, and cytotoxic function.

The best-characterized function of cystatin F is its regulation of protease activity within cytotoxic lymphocytes. In natural killer cells and CD8^+^ T cells, cystatin F colocalizes with cathepsins C, H, and L in cytotoxic granules and regulates the activities of cathepsins C and H, thereby influencing granzyme processing and cytotoxic capacity (Prunk et al., 2020). Although cystatin F can also reduce cathepsin L activity, it does not substantially impair perforin processing, suggesting that its effects on cytotoxic function occur primarily through regulation of the cathepsin–granzyme axis. Cystatin F produced by other cells can also be internalized by cytotoxic lymphocytes through the cation-independent mannose-6-phosphate receptor, reducing cathepsin C activity, granzyme maturation, and cytotoxic function (Prunk et al., 2020; Prunk et al., 2019).

Cystatin F is also expressed in cells of the myeloid lineage. In dendritic cells, its localization changes during maturation, with reported colocalization with cathepsin S in immature cells and with cathepsin L in mature adherent cells (Magister et al., 2012). Cystatin F has additionally been linked to monocyte-to-macrophage differentiation. The *CST7* promoter contains a binding site for CCAAT/enhancer-binding protein-α, a transcription factor involved in myeloid differentiation (Koleva et al., 2012). In phorbol 12-myristate 13-acetate (PMA)-treated U937 cells, differentiation is accompanied by decreased *CST7* expression and cystatin F secretion (Nathanson et al., 2002), and subsequent work confirmed an association between CCAAT/enhancer-binding protein-α and regulation of the *CST7* promoter (Dautović et al., 2018).

Together, these findings establish cystatin F as an immune-enriched, processing-dependent regulator of intracellular cysteine proteases. Its restricted expression, capacity for intercellular transfer, and ability to acquire different protease specificities through sequential processing provide the biochemical framework for understanding its recurrent association with activated neuroimmune cells.

## 3 *CST7* expression across activated neuroimmune populations

Although initially characterized in peripheral immune cells, advances in transcriptomic profiling have substantially expanded the range of neuroimmune populations in which *CST7* expression has been identified in both the central nervous system (CNS) and peripheral immune compartment (Wang et al., 2025). Although *CST7* was initially characterized in cytotoxic lymphocytes and NK cells (Perišić Nanut et al., 2017; Prunk et al., 2019; Prunk et al., 2020), subsequent transcriptomic studies have identified its expression across diverse activated immune populations, including dendritic cells (Magister et al., 2012), macrophages/monocytes (Yao et al., 2025), and microglia (Ma et al., 2011). These observations suggest that *CST7* induction is not restricted to a single lineage but may instead reflect conserved immune programs associated with activation, lysosomal remodeling, and tissue adaptation. In recent years, the emergence of single-cell RNA sequencing (scRNAseq) and spatial transcriptomic technologies has further revealed robust *CST7* upregulation across diverse neurological diseases, where it is repeatedly associated with activated myeloid populations, inflammatory responses, and tissue remodeling. Collectively, these observations position *CST7* as a conserved marker of neuroimmune activation that spans both resident CNS immune cells and infiltrating leukocyte populations.

### 3.1 Microglia and disease-associated neuroimmune states

Microglia are the resident macrophages of the CNS and undergo extensive transcriptional reprogramming in response to infection, injury, aging, and neurodegeneration. Homeostatic microglia are characterized by expression of genes including P2RY12, *TMEM119*, *CX3CR1*, and *SALL1*; however, these signatures are progressively lost as microglia transition toward activated states (Butovsky et al., 2014).

One of the most reproducible features of this transition is the induction of *CST7*. The importance of *CST7* within activated microglial programs first became apparent through large-scale transcriptomic studies of neurodegenerative disease. In their landmark description of disease-associated microglia (DAM), Keren-Shaul and colleagues identified *CST7* among the most highly enriched genes within microglia surrounding amyloid pathology in models of Alzheimer’s disease (Keren-Shaul et al., 2017). *CST7* was co-expressed with genes involved in lysosomal function, lipid metabolism, and phagocytosis, including *APOE*, *CLEC7A*, *LPL*, *CTSD*, and *ITGAX*, suggesting that its induction accompanies acquisition of specialized effector functions rather than generalized inflammatory activation.

Subsequent studies demonstrated that *CST7* expression extends beyond Alzheimer’s disease and is conserved across multiple activated microglial states. Elevated *CST7* expression has since been reported across diverse contexts of neurodegeneration, neuroinflammation, CNS injury, and aging, indicating that its induction is not specific to amyloid pathology, but instead represents a broader feature of neuroimmune activation (Ma et al., 2011; Hammond et al., 2019; Syage et al., 2024). Related populations, including microglial neurodegenerative phenotype (MGnD), activated response microglia (ARM), lipid-associated microglia, and other disease-associated microglial states, consistently exhibit elevated *CST7* expression despite differences in disease context (Krasemann et al., 2017; Sala Frigerio et al., 2019). Together, these independently identified populations suggest that *CST7* forms part of a core transcriptional program associated with neuroimmune activation and tissue adaptation (Daniels et al., 2023).

Importantly, although *CST7* is widely regarded as a hallmark DAM-associated gene, its expression is not unique to microglia. Rather, the prominence of *CST7* within the neurodegeneration literature likely reflects the magnitude and consistency of its induction in activated CNS myeloid populations. This distinction is important, as accumulating evidence indicates that *CST7* expression extends across multiple immune lineages, suggesting that it may represent a broader immune adaptation program rather than a microglia-specific marker.

### 3.2 Aging -associated microglial activation and *CST7* induction

Aging is accompanied by profound changes in immune function throughout the body and is the strongest risk factor for many neurodegenerative diseases. Transcriptomic analyses have consistently demonstrated that aged microglia acquire features resembling disease-associated activation states, including increased expression of genes involved in lysosomal function, lipid metabolism, and inflammatory signaling (Li et al., 2023b; Hickman et al., 2018). Among these changes, *CST7* is prominently expressed within aging-associated microglial activation programs, consistent with broader remodeling of neuroimmune function during aging (Zhang et al., 2022; Morsy et al., 2026).

Studies in aged mice have demonstrated increased *Cst7* expression within activated microglial populations, while single-cell and spatial transcriptomic analyses further indicate that aging-associated microglia increasingly resemble disease-associated states characterized by enhanced lysosomal activity and phagocytic function (Zhang et al., 2022). Importantly, many transcriptional features of aged microglia overlap substantially with DAM and MGnD signatures, suggesting that aging primes microglia toward activation states that become further amplified during neurodegeneration and CNS inflammation (Krasemann et al., 2017; Hammond et al., 2019). The recurrent induction of *CST7* across both aging and disease contexts supports the notion that *CST7* marks a conserved response to chronic tissue stress and neuroimmune activation rather than a disease-specific process.

### 3.3 Cystatin F in cytotoxic lymphocytes

Unlike many genes associated with disease-associated microglia, *CST7* has a well-established history in cytotoxic lymphocyte biology. Long before its emergence in neurodegenerative transcriptomic studies, *CST7* was primarily investigated in cytotoxic immune cells, including NK cells and CD8^+^ T lymphocytes (Perišić Nanut et al., 2017; Kos et al., 2018). In these populations, cystatin F regulates cathepsins involved in granule maturation and cytotoxic effector function, positioning *CST7* as an important modulator of immune-cell activity (Prunk et al., 2019; Perišić Nanut et al., 2017).

Expression of *CST7* has been reported in activated T cells, NK cells, and other hematopoietic immune populations, where it is thought to regulate protease-dependent pathways involved in immune activation and target-cell killing (Hamilton et al., 2008; Perišić Nanut et al., 2017). Although relatively little is known regarding the role of *CST7*-expressing lymphocytes within the CNS, the established biology of *CST7* in cytotoxic immune cells distinguishes it from many canonical DAM-associated genes. Unlike markers such as *TREM2*, *APOE*, or *GPNMB*, *CST7* has a well-established biology in both innate and adaptive immune populations, highlighting its potential role at the intersection of multiple neuroimmune compartments. These observations suggest that *CST7* participates in conserved immune effector programs that are adapted to the specialized functions of distinct immune cell lineages.

This broader expression profile may be particularly relevant in neuroinflammatory disorders where interactions between microglia, infiltrating macrophages, and adaptive immune cells contribute to disease progression. The presence of *CST7* across multiple immune compartments raises the intriguing possibility that *CST7* influences communication between innate and adaptive immune responses during CNS inflammation.

### 3.4 Insights from single-cell and spatial transcriptomics

The widespread adoption of single-cell and spatial transcriptomic technologies has dramatically expanded our understanding of *CST7* expression across the neuroimmune landscape. These approaches have revealed that *CST7* is rarely expressed uniformly across immune populations but instead becomes enriched within discrete cell subsets associated with pathology, tissue remodeling, and inflammatory activation (Keren-Shaul et al., 2017; Masuda et al., 2020).

Across numerous datasets, *CST7*-positive cells consistently co-express genes associated with lysosomal function, phagocytosis, lipid metabolism, and inflammatory signaling, including *APOE, GPNMB, SPP1, LGALS3*, and *CTSD*. Spatial transcriptomic analyses further demonstrate that *CST7*-expressing cells localize to sites of active pathology, including amyloid plaques, demyelinating lesions, and regions of CNS injury, supporting a close association between *CST7* induction and localized neuroimmune activation (Keren-Shaul et al., 2017; Ma et al., 2011; Chen et al., 2020; Masuda et al., 2019; Pettas et al., 2022).

Taken together, single-cell and spatial transcriptomic studies have transformed our understanding of *CST7* from a relatively obscure immune protease inhibitor to a prominent feature of activated neuroimmune states. Although the majority of studies have focused on microglia, accumulating evidence indicates that *CST7* expression extends to cytotoxic lymphocytes and other immune cell populations, suggesting that its induction reflects a conserved response to immune activation, lysosomal remodeling, and tissue stress rather than a microglia-specific phenomenon. At the same time, the biology of *CST7* remains disproportionately characterized in microglia and cytotoxic lymphocytes, while its regulation and function in other myeloid populations remain poorly understood. Determining whether *CST7* serves primarily as a biomarker of immune activation or functions as an active regulator of neuroimmune responses represents a central challenge for the field and provides the framework for the disease- and mechanism-focused studies discussed in the following sections.

## 4 Mechanistic links between cystatin F, lysosomal function, and cell death

The association of *CST7* with diverse activated neuroimmune states raises an important mechanistic question: why is a lysosomal cysteine protease inhibitor associated with such diverse pathological contexts? While the biochemical properties of *CST7* are discussed in Section 2, its consistent transcriptional induction, together with its established biochemical activity, raises the possibility that cystatin F contributes to immune-cell adaptation to the altered lysosomal demands imposed by chronic CNS injury. Accordingly, the following sections examine how *CST7* may intersect with lysosomal homeostasis, antigen presentation, and regulated cell death—three interconnected processes that are increasingly recognized as central to neuroimmune function in health and disease.

A common feature of chronic CNS injury is the increased demand placed on the endosomal-lysosomal system (Malik et al., 2019; Nixon et al., 2000; Somodji and Gowrishankar, 2026). Unlike homeostatic microglia, activated neuroimmune cells continuously engulf apoptotic cells, degenerating myelin, protein aggregates, viral particles, and other cellular debris (Colonna and Butovsky, 2017). Sustained phagocytic activity requires extensive lysosomal remodeling to accommodate increased degradative capacity while maintaining organelle integrity (Lancaster et al., 2021). Consistent with these increased degradative demands, activated microglia undergo extensive lysosomal remodeling accompanied by coordinated transcriptional changes in vesicular trafficking, lipid metabolism, autophagy, and proteolysis (Quick et al., 2023; Somodji and Gowrishankar, 2026; Safaiyan et al., 2021). Rather than representing a generalized activation state, these transcriptional adaptations are increasingly recognized as features of a specialized phagocytic phenotype that equips microglia to sustain debris clearance and tissue remodeling during chronic CNS injury (Safaiyan et al., 2021).

As discussed in Section 2, *CST7* functions as an intracellular inhibitor of multiple lysosomal cysteine proteases. Within the context of chronic neuroinflammation, this biochemical activity raises the possibility that *CST7* contributes to the regulation of lysosomal proteostasis rather than serving solely as a marker of immune activation. Although direct functional evidence in CNS-resident immune cells remains limited, modulation of lysosomal protease activity could influence several processes central to neuroimmune function, including antigen processing, maintenance of lysosomal integrity, and inflammatory signaling (Pišlar et al., 2021; Liu et al., 2019; Di Matteo et al., 2025).

### 4.1 Cystatin F as a regulator of lysosomal proteostasis

Because *CST7* inhibits multiple lysosomal cysteine proteases, its induction during neuroinflammation may have functional consequences extending beyond protein degradation alone. Lysosomal cathepsins regulate numerous aspects of immune cell biology, including degradation of phagocytosed material, turnover of damaged organelles, antigen processing, and maintenance of lysosomal homeostasis (Gallwitz et al., 2024; Stoka et al., 2016). Consequently, dysregulated protease activity has the potential to compromise lysosomal integrity and impair immune cell function (Gallwitz et al., 2024).

Importantly, increased expression of lysosomal genes should not necessarily be interpreted as evidence of increased lysosomal functional capacity. During aging and neurodegenerative disease, lysosomal stress can induce compensatory transcriptional programs that promote lysosomal biogenesis and expression of hydrolytic machinery, even as lysosomes exhibit impaired acidification, reduced degradative capacity, defective membrane repair, and accumulation of undegraded substrates (Steinhauser et al., 2026). Thus, the induction of *CST7* and other lysosomal genes in activated neuroimmune cells may reflect adaptation to increased lysosomal demand or dysfunction rather than simply enhanced lysosomal activity. Whether *CST7* contributes to this compensatory response, helps preserve lysosomal function, or instead influences the development of lysosomal dysfunction, remains unknown.

Based on these observations, *CST7* may function not simply as a brake on cathepsin activity, but rather as a modulator of lysosomal proteolysis, preserving degradative capacity while limiting excessive protease activity during periods of sustained phagocytic stress. Although this hypothesis remains to be directly tested in CNS-resident immune cells, the remarkably consistent induction of *CST7* across neurodegenerative and neuroinflammatory disorders suggests that endogenous regulation of lysosomal proteolysis may represent an important component of the adaptive response to chronic tissue injury.

### 4.2 Potential implications for antigen presentation

The endosomal-lysosomal system serves not only as the primary degradative compartment of professional phagocytes but also as the site where internalized antigens are processed for presentation on major histocompatibility complex class II (MHC II) molecules. Lysosomal cysteine cathepsins contribute to multiple steps of this pathway, including degradation of internalized proteins, proteolytic processing of the invariant chain (CD74), and generation of peptides for MHC II loading (Hsing and Rudensky, 2005). Among these, cathepsin S is the principal invariant chain protease in professional antigen-presenting cells, while cathepsin L contributes in a cell type-dependent manner (Riese et al., 1996; Hsieh et al., 2002). Importantly, antigen processing is not mediated by a single protease but rather by the coordinated activity of multiple lysosomal cathepsins whose relative contributions vary according to cell type, activation state, and inflammatory context. Consequently, efficient antigen presentation depends on tightly regulated lysosomal proteolysis rather than maximal protease activity.

Although cathepsin C is the best-characterized target of *CST7*, *CST7* also inhibits several additional lysosomal cysteine cathepsins, including cathepsins H, L, and S (Section 2). Thus, alterations in *CST7* expression could theoretically influence the balance of lysosomal protease activity required for antigen processing rather than regulating a single protease in isolation. Because excessive proteolysis may destroy antigenic epitopes whereas insufficient proteolysis may impair peptide generation, endogenous regulators of cathepsin activity may be important for maintaining optimal antigen presentation during chronic immune activation (Villadangos and Ploegh, 2000).

Microglia and infiltrating macrophages upregulate MHC II expression during aging, viral encephalitis, multiple sclerosis, and numerous neurodegenerative disorders, reflecting an enhanced capacity to engage adaptive immune responses (Safaiyan et al., 2021; Wheeler et al., 2018; Mangale et al., 2020; Jordão et al., 2019). Whether *CST7* contributes to this transition by modulating lysosomal protease activity remains unknown. To date, no study has directly demonstrated that manipulation of *CST7* alters antigen processing or MHC II presentation in CNS-resident immune cells. However, its reproducible induction in MHC II-expressing neuroimmune populations, together with its established role in regulating lysosomal proteases, provides a plausible mechanistic link between lysosomal remodeling and adaptive immune activation. Determining whether *CST7* influences peptide processing, phagosome maturation, or antigen presentation efficiency represents an important direction for future investigation.

### 4.3 Lysosomal stress and inflammatory cell death

Chronic activation of the endosomal-lysosomal system is increasingly recognized as a driver of inflammatory signaling (Lee et al., 2023). Persistent accumulation of protein aggregates, oxidized lipids, cholesterol crystals, or damaged cellular components can overwhelm lysosomal degradative capacity, leading to lysosomal membrane permeabilization and release of cathepsins into the cytosol (Amaral et al., 2023; Aits and Jäättelä, 2013). Among these, cytosolic cathepsin B has been most strongly implicated in the activation of the NLRP3 inflammasome, while lysosomal cathepsins more broadly can promote apoptotic signaling through cleavage of pro-apoptotic substrates, linking lysosomal dysfunction to both pyroptotic and apoptotic cell death (Hoegen et al., 2011; Bruchard et al., 2013; Rintahaka et al., 2011; Halle et al., 2008).

Within this framework, *CST7* occupies an intriguing position as an endogenous regulator of lysosomal protease activity. Although *CST7* primarily inhibits cathepsins C, H, L, and S rather than cathepsin B (Section 2), alterations in lysosomal protease activity may nevertheless influence the broader proteolytic environment that governs lysosomal homeostasis and downstream inflammatory signaling. Importantly, these connections are inferred from the broader biology of lysosomal cathepsins rather than from direct evidence linking cystatin F to inflammasome activation, pyroptosis, or apoptosis in CNS cells. Whether *CST7-*mediated regulation of lysosomal proteases modifies susceptibility to lysosomal membrane permeabilization, or downstream inflammatory cell-death pathways, therefore remains unknown. *CST7* could theoretically modulate these processes by altering the lysosomal proteolytic environment, but direct *CST7-*specific studies will be required to establish whether such mechanisms operate during CNS disease. Addressing these questions through cell type-specific genetic models will be essential to determine whether *CST7* actively protects against lysosomal dysfunction or simply serves as a marker of cells experiencing increased lysosomal demand.

## 5 Cystatin F across neurodegenerative disease

Cystatin F induction is observed across several neurodegenerative diseases, particularly within activated or disease-associated myeloid populations. Although much of the mechanistic evidence comes from animal models, the consistency of this response across distinct pathological contexts suggests that *CST7* is a shared component of neurodegeneration-associated immune remodeling rather than a marker unique to a single disorder. The following sections examine the evidence for cystatin F in Alzheimer’s disease, amyotrophic lateral sclerosis (ALS), and Parkinson’s disease, with attention to the strength of the human evidence, its cellular source, and its potential functional significance. A comparative overview of *CST7* expression and proposed roles across these and other neurological disorders discussed throughout this review is provided in Table 1.

**TABLE 1:** Summary of *CST7*/Cystatin F expression across CNS disease contexts.

Disease/Condition	Human evidence	Representative mouse model(s)	Major *CST7*-expressing cell population(s)	Proposed role of cystatin F	Evidence type	Key references
Neurodegenerative disorders						
Aging	Detected in aging human microglia by transcriptomic studies	Naturally aged mice	Activated microglia	Adaptation to increased lysosomal demand, phagocytosis, immune activation	Transcriptomic evidence	Hammond et al., 2019; Masuda et al., 2019; Zhang et al., 2022; Morsy et al., 2026
Alzheimer’s disease	Inconsistently detected in human AD microglia by snRNA-seq	5xFAD, APP/PS1, APP^NL−G-F^	Disease-associated microglia	Lysosomal regulation during amyloid clearance and chronic inflammation	Protein evidence	Keren-Shaul et al., 2017; Krasemann et al., 2017; Daniels et al., 2023; Zhou et al., 2020
Parkinson’s disease	No convincing human evidence	AAV-α-synuclein; MPTP	Activated microglia	Response to protein aggregation and lysosomal stress	Protein evidence	Schonhoff et al., 2023; Leem et al., 2023
Amyotrophic lateral sclerosis	No convincing human evidence	SOD1^G93A^; rNLS8 (TDP-43)	Activated microglia	Chronic neuroimmune activation and debris clearance	Transcriptomic evidence	Keren-Shaul et al., 2017; Sobue et al., 2021; Hunter et al., 2021
Neuroinflammatory disorders						
Multiple sclerosis	Detected in microglia and macrophages at active demyelinating plaque edges in human MS tissue	EAE; cuprizone; Plp1 overexpression	Microglia, infiltrating macrophages	Myelin debris clearance, inflammatory regulation, remyelination	Transcriptomic evidence	Jordão et al., 2019; Masuda et al., 2019; Liang et al., 2016; Syage et al., 2023
Viral encephalitis	No convincing human evidence	JHMV	Microglia, infiltrating myeloid cells, cytotoxic lymphocytes	Regulation of antiviral neuroinflammation and tissue damage	Functional evidence (global knockout)	Syage et al., 2020; Syage et al., 2024
CNS injury and repair						
Traumatic brain injury	No convincing human evidence	Controlled cortical impact	Activated microglia	Injury-associated phagocytic remodeling	Transcriptomic evidence	Pettas et al., 2022; Arneson et al., 2018
Spinal cord injury	No convincing human evidence	Contusion SCI	Activated microglia	Debris clearance and chronic inflammatory remodeling	Transcriptomic evidence	Hakim et al., 2021
Ischemic stroke	No convincing human evidence	MCAO	Activated microglia	Clearance of necrotic tissue and inflammatory resolution	Transcriptomic evidence	Zheng et al., 2022

Evidence type indicates the most direct *CST7*-specific evidence available for each disease context: transcriptomic evidence, *CST7/Cst7* expression demonstrated at the transcript level; protein evidence, cystatin F expression demonstrated at the protein level; functional evidence, *CST7/Cst7* directly manipulated genetically or pharmacologically. Abbreviations: AD, Alzheimer’s disease; AAV, adeno-associated virus; APP, amyloid precursor protein; EAE, experimental autoimmune encephalomyelitis; JHMV, JHM strain of mouse hepatitis virus; MCAO, middle cerebral artery occlusion; MPTP, 1-methyl-4-phenyl-1,2,3,6-tetrahydropyridine; MS, multiple sclerosis; SCI, spinal cord injury; snRNA-seq, single-nucleus RNA sequencing; TDP-43, TAR DNA-binding protein 43.

### 5.1 Cystatin F in Alzheimer’s disease

Among neurodegenerative disorders, Alzheimer’s disease (AD) provides the strongest evidence linking *CST7* to disease-associated microglial activation. Following the original identification of disease-associated microglia (DAM), *Cst7* emerged as one of the most consistently upregulated genes across multiple murine models of amyloid pathology and has since become a canonical component of the DAM transcriptional program (Keren-Shaul et al., 2017). Importantly, *Cst7* induction is not unique to AD, as increased expression is also observed in SOD1-G93A mice and other neurodegenerative models, supporting the concept that cystatin F participates in a conserved microglial response to chronic neurodegeneration rather than an amyloid-specific program (Keren-Shaul et al., 2017).

Subsequent studies demonstrated that cystatin F expression is highly enriched within plaque-associated microglia. In APP/PS1 mice, cystatin F-positive microglia localize around amyloid plaques while remaining largely absent from homeostatic microglia distant from pathology (Ofengeim et al., 2017). Similar observations have been reported in the App^NL−G-F^ knock-in model, where *Cst7* expression is restricted to IBA1-positive plaque-associated microglia and is not detected in border-associated macrophages or other CNS macrophage populations (Daniels et al., 2023). Together, these studies indicate that *Cst7* induction accompanies the transition from homeostatic to activated plaque-associated microglial states.

Although most work has focused on amyloid pathology, recent evidence suggests that *Cst7* is also induced in response to specific forms of tau pathology. Arribas-Gomez et al. reported robust *Cst7* upregulation in mice expressing fibrillar P301L tau, whereas mice expressing the A152T tau variant exhibited a predominantly astrocytic transcriptional response (Arribas Gomez et al., 2025). Because fibrillar tau is associated with a stronger DAM-like phenotype, these findings suggest that cystatin F induction may reflect the nature of the underlying proteinopathy rather than simply the presence of tau itself. Interestingly, cystatin F is one of the few endogenous inhibitors of legumain, a lysosomal protease capable of generating aggregation-prone tau fragments through cleavage at Asn368 (Zhang et al., 2014). Whether cystatin F regulates tau processing *in vivo*, however, remains unknown.

Translation of these findings to human AD has proven considerably less straightforward. Several single-nucleus RNA sequencing studies have reported that, despite robust induction of many DAM-associated pathways, *CST7* is inconsistently detected in human AD microglia (Mathys et al., 2019; Zhou et al., 2020; Gerrits et al., 2021; Sobue et al., 2021). This discrepancy has generated considerable discussion within the field. Proposed explanations include differences in disease stage, species-specific transcriptional programs, and technical limitations of single-nucleus sequencing, which may underrepresent activation-associated transcripts compared with whole-cell sequencing (Thrupp et al., 2020). In addition, sex-dependent regulation has been proposed following observations in murine models, although this has not yet been confirmed in human tissue (Daniels et al., 2023). Importantly, the absence of robust *CST7* detection in transcriptomic datasets does not necessarily imply that the underlying signaling pathway is absent. Indeed, TREM2 overexpression is sufficient to induce cystatin F expression in cultured human microglia-like cells, indicating that the pathway remains functional under appropriate conditions (Popescu et al., 2023).

Beyond its value as a marker of activated microglia, several studies have begun to investigate whether cystatin F actively contributes to AD pathogenesis. Early work identified RIPK1 signaling as an upstream regulator of *Cst7* expression in APP/PS1 mice, and both pharmacological and genetic inhibition of RIPK1 reduced cystatin F-positive microglia while improving microglial amyloid clearance (Ofengeim et al., 2017). *In vitro* overexpression studies further demonstrated that elevated cystatin F inhibits cathepsin C activity and alters lysosomal function, leading to the proposal that excessive cystatin F limits microglial degradative capacity.

More recent genetic studies have refined this model. Using App^NL-G-F^ mice crossed with *Cst7* knockout animals, Daniels et al. demonstrated that cystatin F regulates microglial responses in a sexually dimorphic manner (Daniels et al., 2023). Female knockout mice exhibited increased lysosomal activity and enhanced microglial phagocytosis, whereas males showed reduced inflammatory activation with comparatively minor effects on amyloid burden. Interestingly, cystatin F deficiency did not alter intracellular Aβ degradation, suggesting that its primary effect may be modulation of phagocytic uptake rather than downstream lysosomal proteolysis. These findings suggest that cystatin F influences multiple aspects of microglial activation that extend beyond simple inhibition of lysosomal cathepsins.

An additional layer of complexity has recently emerged from studies of peripheral immunity. Li et al. reported increased *CST7* expression in circulating monocytes from patients with sporadic AD and demonstrated that secreted dimeric cystatin F directly binds amyloid-β, thereby inhibiting monocyte-mediated phagocytic clearance (Li et al., 2024). Monocyte-specific overexpression of human *CST7*, or peripheral administration of purified cystatin F, increased plaque burden and worsened cognitive impairment in APP/PS1 mice, suggesting that peripheral cystatin F may influence AD progression through mechanisms distinct from those operating in microglia. Conversely, higher CSF cystatin F concentrations have been associated with slower tau accumulation and reduced cognitive decline in patients with sporadic AD (Pereira et al., 2022). Together, these findings raise the possibility that the biological effects of cystatin F depend on cellular source, structural form, and disease context.

Taken together, current evidence establishes cystatin F as a robust feature of activated microglia in murine AD models, while its role in human disease remains less clearly defined. Although growing functional evidence supports effects on phagocytosis, lysosomal biology, and immune activation, these findings do not yet converge on a single pathogenic or protective role. Instead, the available data suggest that cystatin F functions are likely context dependent, varying according to cell type, sex, disease stage, and tissue compartment. Defining these context-specific functions—and determining whether they extend to human disease—will be essential for understanding whether cystatin F is simply a marker of activated microglia or a *bona fide* regulator of AD pathogenesis.

### 5.2 Amyotrophic lateral sclerosis (ALS)

*Cst7* induction is not confined to microglia in Alzheimer’s disease models. The single-cell study that originally defined the disease-associated microglia (DAM) signature identified *Cst7* as a canonical DAM marker and noted its induction across neurodegenerative contexts, including the SOD1-G93A mouse model of amyotrophic lateral sclerosis (ALS) (Keren-Shaul et al., 2017). This observation was subsequently supported by Sobue et al. (2021), who compared RNA-seq profiles of microglia isolated from three neurodegenerative mouse models—including SOD1-G93A mice—and found broad induction of DAM-associated genes in all three models (Sobue et al., 2021).

*Cst7* induction has also been observed in an ALS-relevant TDP-43 proteinopathy model. Longitudinal RNA-seq of cortical and spinal cord microglia from rNLS8 mice, which conditionally express cytoplasmic human TDP-43 lacking a nuclear localization signal, identified *Cst7* among a group of DAM-associated genes strongly upregulated during late disease and recovery (Hunter et al., 2021). This finding is important because TDP-43 pathology occurs in approximately 97% of ALS cases, whereas SOD1 mutations account for only a small subset of familial ALS (Suk and Rousseaux, 2020). Nevertheless, rNLS8 remains an experimental overexpression model and should be regarded as reproducing selected features of ALS-associated TDP-43 pathology rather than the full human disease.

A distinctive feature of the rNLS8 model is its reversibility. Removing doxycycline induces expression of cytoplasmic human TDP-43 and produces progressive neurodegeneration, whereas re-administering doxycycline suppresses the transgene and initiates functional and pathological recovery (Hunter et al., 2021). *Cst7* expression exhibited different temporal patterns in cortical and spinal cord microglia. In cortical microglia, *Cst7* induction was detectable during early disease, increased substantially during late disease, and remained elevated during recovery. In spinal cord microglia, significant induction was not detected during early disease but emerged during late disease and became more pronounced during recovery (Hunter et al., 2021).

The persistence of *Cst7* expression after TDP-43 suppression indicates that it accompanies the recovery-associated microglial response rather than simply tracking ongoing transgene expression. In an earlier study using the same rNLS8 model, pharmacological suppression of microgliosis during early recovery impaired neuronal TDP-43 clearance and prevented full motor recovery, supporting a beneficial role for the broader microglial response during this phase (Spiller et al., 2018). However, that study did not manipulate or otherwise isolate *CST7* function. It therefore remains unknown whether *CST7* promotes recovery, constrains it, or simply marks the recovery-associated microglial state.

Despite consistent *Cst7* induction in mouse ALS models driven by both mutant SOD1 and TDP-43 pathology, direct human evidence remains limited. To our knowledge, *CST7* has not been specifically validated in human ALS postmortem tissue or biofluids as a disease-associated marker or biomarker. Dedicated studies are therefore needed to determine whether the *CST7* response observed in mouse microglia is conserved in human ALS, whether it varies across disease stages or molecular subtypes, and whether *CST7* has value as a tissue or fluid biomarker.

### 5.3 Parkinson’s disease

Evidence connecting cystatin F with Parkinson’s disease (PD) has expanded but remains less developed than the evidence in Alzheimer’s disease or mouse models of ALS. Schonhoff et al. identified disease-associated microglial and border-associated macrophage states in an adeno-associated virus (AAV)–α-synuclein mouse model of PD using single-cell RNA sequencing (Schonhoff et al., 2023). The disease-associated microglial (DAM) population expressed *Cst7* together with other DAM-associated genes, including *Apoe* and *Lpl*. However, the study’s principal functional findings concerned the role of border-associated macrophages in antigen presentation and neuroinflammation, and it did not establish a functional role for *Cst7* itself (Schonhoff et al., 2023).

Additional evidence comes from a subacute 1-methyl-4-phenyl-1,2,3,6-tetrahydropyridine (MPTP) mouse model of PD. MPTP treatment increased cystatin F protein in substantia nigra tissue alongside microglial activation, reactive astrogliosis, and increased expression of inflammatory mediators including *iNOS*, *Il-1β*, and *Tnf-α* (Leem et al., 2023). Treatment with necrosulfonamide, a pharmacological inhibitor used to disrupt mixed lineage kinase domain-like protein (MLKL)-associated signaling, reduced cystatin F expression as well as these inflammatory and pathological responses. Because cystatin F was measured at the tissue level and multiple aspects of MPTP pathology changed concurrently, these findings do not by themselves demonstrate that *Cst7* was induced exclusively in microglia or that it was a direct downstream target of MLKL (Leem et al., 2023).

These observations can be compared with findings in an amyloid precursor protein/presenilin 1 (APP/PS1) mouse model of Alzheimer’s disease, in which genetic or pharmacological inhibition of receptor-interacting protein kinase 1 (RIPK1) reduced microglial *Cst7* expression (Ofengeim et al., 2017). Importantly, that study characterized RIPK1-dependent *Cst7* induction as part of an inflammatory, non-cell-death transcriptional program rather than demonstrating that *Cst7* was induced through canonical RIPK1, receptor-interacting-protein kinase 3 (RIPK3), and MLKL necroptosis (Ofengeim et al., 2017). The AD and MPTP findings therefore suggest that *Cst7* expression may be responsive to signaling associated with RIPK1 and MLKL, respectively, but they do not establish a conserved RIPK1–RIPK3–MLKL pathway that directly drives *Cst7* induction across the two diseases.

The functional significance of *Cst7* in PD consequently remains unresolved. No *Cst7* loss-of-function experiments in PD models have yet determined whether *Cst7* affects α-synuclein processing, inflammatory signaling, lysosomal function, or dopaminergic neuron survival. Likewise, *Cst7* has not been specifically validated as a biomarker in PD cerebrospinal fluid or plasma. Future studies will need to establish the cellular source of *CST7* in human PD tissue, identify the signaling mechanisms responsible for its induction, and determine whether *CST7* contributes to pathology or primarily marks a disease-associated myeloid state.

## 6 Cystatin F in CNS inflammation

Inflammatory disorders of the CNS arise from diverse etiologies, including autoimmune disease, viral infection, and sterile tissue injury (Li et al., 2023a; Shichita et al., 2012; Cheng et al., 2023). Although these conditions differ substantially in their initiating mechanisms, each requires coordinated interactions among resident glial cells, recruited leukocytes, and damaged neural tissue. These responses support pathogen control, clearance of cellular and myelin debris, and tissue repair, but can also amplify secondary injury when inflammatory activity persists or becomes dysregulated. *CST7* has emerged as a recurring component of these responses, particularly in immune populations undergoing substantial lysosomal, phagocytic, or cytotoxic remodeling (Hammond et al., 2019). Importantly, studies of CNS inflammation have begun to move beyond the descriptive identification of *CST7*-expressing cells and provide evidence that cystatin F can actively influence inflammatory disease severity and white matter repair.

### 6.1 Immune-mediated demyelinating disease

Multiple sclerosis (MS) is a chronic inflammatory demyelinating disease characterized by autoreactive lymphocyte infiltration, myelin destruction, axonal degeneration, and progressive neurological disability (Compston and Coles, 2008). Autoreactive lymphocytes initiate and sustain inflammatory responses, while microglia and monocyte-derived macrophages participate in antigen presentation, cytokine and chemokine production, removal of damaged myelin, and remodeling of the lesion environment. Because myelin is lipid-rich and resistant to degradation, its uptake places considerable metabolic and lysosomal demands on phagocytic cells (Yen and Yu, 2023). The balance between efficient debris clearance and excessive inflammatory proteolysis is therefore likely to influence whether lesions progress toward continued tissue injury or successful repair.

Cystatin F was initially linked to demyelinating disease through its spatial and temporal association with active myelin damage and repair (Jordão et al., 2019; Masuda et al., 2019). In human MS tissue, cystatin F has been detected in microglia and macrophages at the leading edges of demyelinating plaques, regions in which myelin breakdown and immune-cell activation are actively occurring (Masuda et al., 2019). Experimental models similarly demonstrated that microglial *Cst7* expression increases during demyelination accompanied by remyelination and can be induced by the phagocytosis of myelin debris (Krasemann et al., 2017; Syage et al., 2023). These findings distinguished *Cst7* from a nonspecific marker of chronic gliosis and instead associated its expression with dynamic areas of white matter turnover. The localization of cystatin F within both experimental demyelinating lesions and human MS plaques further suggested that its induction may represent a conserved response to myelin injury.

Evidence from several complementary models has strengthened this association. *Cst7* expression has been reported in experimental autoimmune encephalomyelitis (EAE–the prototypic model of MS) (Jordão et al., 2019), toxin-induced demyelination (Krasemann et al., 2017), and genetic models of chronic myelin disruption including proteolipid protein 1 (*Plp1)* overexpression (Shimizu et al., 2017). In these settings, *Cst7* is commonly detected alongside changes in cathepsin expression and in myeloid populations involved in myelin uptake and lesion remodeling (Ma et al., 2007). Importantly, these models initiate demyelination through distinct mechanisms. EAE is driven predominantly by autoreactive adaptive immunity, whereas cuprizone induces oligodendrocyte injury and demyelination without requiring myelin-specific T-cell priming (Lassmann and Bradl, 2017). In contrast, *Plp1-*overexpressing mice develop chronic myelin instability resulting from a primary genetic defect in myelin (Shimizu et al., 2017). The induction of *Cst7* across these mechanistically distinct paradigms indicates that its expression is not uniquely coupled to autoimmunity, but more broadly to the cellular response to myelin damage and repair.

Studies manipulating the cystatin F–cathepsin axis have also provided evidence that this response is functionally relevant. In a chronic demyelination model produced by overexpression of *Plp1,* the balance between cystatin F and cathepsin C was implicated in the control of remyelination (Ma et al., 2011; Shimizu et al., 2017). In the cuprizone model, loss of cystatin F enhanced cathepsin C activity and aggravated demyelination, accompanied by increased expression of inflammatory mediators (Liang et al., 2016). These observations support a model in which *Cst7* restrains excessive cathepsin-dependent inflammation during white matter injury. They also suggest that *Cst7* induction may constitute a compensatory response intended to preserve a permissive environment for repair rather than simply reflecting the abundance of activated microglia.

Despite the reproducibility of *Cst7* expression across demyelinating models, these studies were largely descriptive and provided limited insight into its biological function. An important advance came from the application of scRNAseq to the JHM strain of mouse hepatitis virus (JHMV) model, which recapitulates many features of chronic, immune-mediated demyelination. In chronically demyelinated spinal cord lesions, *Cst7* was identified as one of the most highly enriched genes within a disease-associated microglial subset (MG3) (Syage et al., 2020). In addition to *Cst7,* these microglia expressed genes associated with lipid metabolism, phagocytosis, and tissue repair, including *Igf1* ***,*** *Lpl* ***,*** *Apoe* ***,*** *Spp1, and Trem2,* and gene ontology analyses demonstrated enrichment of pathways involved in oligodendrocyte differentiation and remyelination. Together, these findings associated *Cst7* with a microglial state characterized by reparative and remyelination-associated programs rather than inflammation alone.

To determine whether *Cst7* actively regulates inflammatory demyelination rather than simply serving as a marker of inflammatory activation, its function was subsequently examined using global *Cst7-*deficient mice (Syage et al., 2024). The biphasic JHMV model, which transitions from acute viral encephalitis to chronic inflammation characterized by persistent viral RNA and antigen, spinal cord demyelination, and incomplete remyelination, enables effects on antiviral immunity to be distinguished from those on chronic neuroinflammation and tissue repair (Bergmann et al., 2006; Lane and Hosking, 2010).

Loss of *Cst7* did not significantly alter viral titers during acute infection or viral RNA levels during chronic disease, and early accumulation of total and virus-specific T cells was largely preserved, indicating that cystatin F was not essential for antiviral immunity or control of JHMV replication. In contrast, *Cst7-*deficient mice developed more severe chronic neurological disease, with increased spinal cord demyelination, axonal damage, and impaired remyelination. Electron microscopy confirmed a higher proportion of demyelinated axons and fewer remyelinated axons in infected knockout mice, demonstrating that *Cst7* deficiency affected both white matter injury and subsequent repair (Syage et al., 2024).

This worsened pathology was accompanied by heightened inflammatory activity. Early in chronic disease, *Cst7-*deficient mice exhibited increased accumulation of microglia and CD8^+^ T cells in the spinal cord. At a later time point, single-cell RNA sequencing revealed elevated expression of *Cxcl9* across microglia, macrophages, and inflammatory monocytes, together with increased *Cxcl10* and *Ccl2* in monocytes and increased *Tnf* in microglia and macrophages. Interferon-γ response programs were also enhanced in myeloid and lymphoid populations, while effector and cycling CD8^+^ T cells displayed increased *Ifng* expression and proliferating CD8^+^ T cells showed elevated *Prf1*. These findings suggested that cystatin F limits a feed-forward inflammatory circuit involving myeloid chemokine production, T-cell recruitment or activation, and interferon-driven white matter injury (Syage et al., 2024).

Notably, impaired remyelination in *Cst7*-deficient mice was not accompanied by a broad loss of canonical repair-associated transcriptional programs or an obvious reduction in genes associated with myelin phagocytosis. Instead, microglia and monocyte/macrophage populations showed increased expression of several lipid-handling and phagocytosis-associated genes, potentially reflecting the greater burden of damaged myelin in knockout animals. The phenotype therefore appears unlikely to result simply from an inability of *Cst7*-deficient myeloid cells to internalize debris. A more plausible interpretation is that excessive inflammatory signaling and immune-cell activation create an environment that remains hostile to oligodendrocyte survival, differentiation, or remyelination despite continued engagement of phagocytic pathways.

Together, these studies establish *Cst7* as more than a histological or transcriptional correlate of demyelination. Across toxin, genetic, autoimmune, and virus-induced models, cystatin F is positioned at the interface of myelin clearance, inflammatory regulation, and repair. The JHMV findings further indicate that *Cst7* can limit chronic neuroinflammatory injury without compromising antiviral control, suggesting that its protective effects are separable from the immune mechanisms required for pathogen restriction. An important limitation of this study, however, is that global *Cst7* deletion cannot identify which cellular source is responsible for the observed phenotype. *Cst7* produced by microglia could regulate myeloid cathepsin activity and chemokine expression, whereas lymphocyte-derived *Cst7* could restrain cytotoxic effector function in an autocrine or paracrine manner. Cell type-specific genetic studies will therefore be necessary to distinguish the contributions of resident microglia, recruited myeloid cells, and cytotoxic lymphocytes to *Cst7*-dependent regulation of demyelination and remyelination.

### 6.2 Viral encephalitis

Viral encephalitis presents a fundamentally different immunological challenge than autoimmune demyelination. Rather than responding to sterile tissue injury, resident CNS immune cells must simultaneously restrict viral replication, coordinate recruitment of antiviral lymphocytes, and minimize immunopathology within the highly vulnerable neural parenchyma (Hosking et al., 2009; Syage et al., 2020; Mangale et al., 2020). Successful viral control therefore depends upon precise coordination between innate and adaptive immune responses.

Microglia are central orchestrators of this process (Mangale et al., 2020). Following infection, they undergo extensive transcriptional reprogramming characterized by enhanced interferon signaling, increased expression of major histocompatibility complex molecules, and expansion of lysosomal and phagocytic pathways necessary for clearance of infected cells and presentation of viral antigens (Syage et al., 2023). Across multiple models of viral neuroinflammation, including infections caused by neurotropic coronaviruses, *CST7* has been identified among the genes consistently induced within activated myeloid populations (Syage et al., 2023). Although the precise magnitude of induction varies between viral pathogens and experimental systems, these studies collectively suggest that *CST7* forms part of a conserved transcriptional response accompanying antiviral activation of CNS myeloid cells.

Compared with autoimmune disease, viral infection imposes unique demands on lysosomal biology (Richards et al., 2022). In addition to degrading apoptotic debris, activated microglia must continuously process viral proteins for antigen presentation while eliminating infected cellular material. Cathepsins play essential roles throughout these processes, participating in endosomal protein degradation, MHC class II peptide loading, and regulation of inflammatory signaling pathways (Zhao et al., 2024; Scarcella et al., 2022). As an endogenous inhibitor of several lysosomal cathepsins, *CST7* occupies a potentially important regulatory position within this network. It is therefore tempting to speculate that *CST7* functions to prevent excessive proteolysis during sustained antiviral responses, thereby preserving lysosomal integrity while maintaining effective antigen processing. However, direct experimental evidence supporting this hypothesis is currently lacking.

Another notable feature of viral encephalitis is the dynamic interaction between activated microglia and infiltrating T lymphocytes (Syage et al., 2023; Templeton and Perlman, 2008). Efficient antigen presentation is required to sustain antiviral T-cell responses within infected CNS tissue, yet excessive inflammatory activation contributes directly to neuronal injury (Savarin et al., 2012; Phares et al., 2012). Whether *CST7* influences this balance through modulation of lysosomal protease activity represents an intriguing but unanswered question. Future studies employing genetic manipulation of *CST7* in experimental viral encephalitis models will therefore be important for determining whether this molecule contributes to antiviral immunity, regulation of neuroinflammation, or resolution of tissue injury following infection.

### 6.3 CNS injury

Sterile CNS injuries, including traumatic brain injury (TBI), spinal cord injury (SCI), and ischemic stroke, initiate inflammatory responses that are distinct from infection or autoimmunity yet converge upon many of the same cellular programs (Chen and Nuñez, 2010). Within hours of injury, resident microglia become activated, peripheral myeloid cells infiltrate damaged tissue, and large quantities of apoptotic cells, disrupted myelin, and extracellular debris must be removed before tissue repair can proceed (Swarts and Brennan, 2025; Ahn et al., 2018).

Single-cell transcriptomic studies have consistently demonstrated robust induction of *CST7* following TBI, SCI, and cerebral ischemia (Pettas et al., 2022; Hakim et al., 2021; Arneson et al., 2018; Zheng et al., 2022). In each of these settings, *CST7* is enriched within highly phagocytic microglial and macrophage populations that simultaneously express genes associated with lysosomal biogenesis, lipid metabolism, extracellular matrix remodeling, and tissue repair. Although the precise composition of these injury-associated populations varies depending on the timing and nature of the insult, their transcriptional programs overlap substantially with those described in both neurodegenerative disease and autoimmune demyelination (Keren-Shaul et al., 2017; Hammond et al., 2019).

The reproducibility of *CST7* induction across diverse injury models is particularly noteworthy because these conditions lack chronic protein aggregation and, unlike viral encephalitis, are not driven by persistent pathogen recognition (Lane and Hosking, 2010). Instead, the common denominator appears to be the extensive requirement for degradation of damaged tissue and restoration of CNS homeostasis (Lloyd and Miron, 2019; Hakim et al., 2021). Clearance of myelin fragments, necrotic cellular debris, and apoptotic cells places extraordinary demands upon lysosomal proteolytic pathways, providing a plausible biological explanation for coordinated induction of *CST7* alongside multiple cathepsins and other lysosomal genes (Safaiyan et al., 2021; Colonna and Butovsky, 2017).

Whether cystatin F contributes directly to tissue repair following CNS injury remains unknown. Controlled proteolysis is essential for efficient debris clearance and subsequent resolution of inflammation, whereas excessive cathepsin activity has been implicated in lysosomal membrane permeabilization, inflammasome activation, and secondary neuronal damage (Safaiyan et al., 2021; Hakim et al., 2021; Liang et al., 2016; Shimizu et al., 2017). Cystatin F may therefore function as an endogenous mechanism that fine-tunes lysosomal protease activity during tissue repair, limiting detrimental protease-mediated injury without compromising phagocytic function. Testing this hypothesis will require cell type-specific genetic approaches capable of distinguishing the contributions of resident microglia from infiltrating macrophages throughout the different phases of injury and repair.

Whether *CST7* functions primarily to facilitate lysosomal homeostasis, restrain excessive protease activity, or coordinate inflammatory resolution following CNS injury remains an important area of investigation. Elucidating these mechanisms will be critical for determining whether *CST7* represents simply a biomarker of injury-associated neuroimmune activation or an active regulator of tissue repair.

## 7 Is CST7 a biomarker or a functional regulator? Current evidence and outstanding questions

One of the most striking observations to emerge from the literature is the disconnect between the prominence of *CST7* in neuroimmune datasets and the limited understanding of its functional role. As discussed in Section 3, across neurodegenerative diseases, CNS inflammation, aging, and tissue injury, *CST7* is consistently among the most highly upregulated genes within activated immune cell populations. Consequently, it has become widely adopted as a marker of disease-associated microglia and other activated myeloid cells. Despite its frequent inclusion in transcriptional signatures, relatively few studies have moved beyond descriptive analyses to investigate whether *CST7* actively contributes to neuroimmune function.

This distinction is important because transcriptomic studies alone cannot determine whether *CST7* is simply a marker of activated neuroimmune cells or whether it contributes directly to their function. Consequently, *CST7* has largely been interpreted as one component of broader activation programs, often alongside *SPP1*, *APOE*, *GPNMB*, and *LGALS3*, rather than as a potential regulator of disease processes (Keren-Shaul et al., 2017; Krasemann et al., 2017). While these studies have firmly established *CST7* as a reproducible marker of neuroimmune activation, they provide little insight into why its expression is so consistently induced, what signals regulate its expression, or whether it actively influences disease progression.

The preceding sections of this review nevertheless provide a rationale for investigating whether a purely descriptive interpretation is sufficient. Unlike many genes identified through unbiased transcriptomic analyses, *CST7* encodes a protein with a well-defined biochemical function. As highlighted in Section 2, cystatin F is a selective endogenous inhibitor of lysosomal cysteine cathepsins, proteases that regulate antigen processing, lysosomal homeostasis, phagosome maturation, and inflammatory signaling. These pathways are central to the biology of activated microglia and infiltrating myeloid cells during CNS disease. The repeated induction of *CST7* across diverse pathological conditions therefore raises the possibility that its expression reflects an adaptive regulatory response rather than simply a transcriptional consequence of immune activation.

At present, however, direct functional evidence within the CNS remains limited. While the biochemical functions of cystatin F are well characterized, relatively little is known about how these activities influence neuroimmune cell behavior during disease. Few studies have manipulated *CST7* expression *in vivo*, leaving its physiological role in regulating CNS inflammation largely unresolved.

Addressing these questions will require moving beyond descriptive transcriptomics toward mechanistic investigation. An important priority is determining how *CST7* activity is regulated during CNS disease and how its established biochemical functions shape neuroimmune cell behavior *in vivo.* Defining the upstream signals that induce *CST7*, the downstream cathepsin pathways it modulates, and the cell type-specific consequences of its expression will be essential for understanding its contribution to neuroimmune responses. Equally important is determining the functional consequences of *CST7* expression across different immune cell populations. Although most studies have focused on microglia, *CST7* is expressed by multiple innate and adaptive immune cell types, suggesting that its biological functions may extend beyond any single population. Emerging conditional genetic models, together with single-cell and spatial technologies, now provide an opportunity to define these cell type-specific roles with unprecedented resolution.

Another critical question is whether *CST7* ultimately serves protective or pathogenic functions during disease progression. As an endogenous inhibitor of lysosomal cysteine proteases, *CST7* may preserve lysosomal homeostasis and restrain excessive protease activity during chronic inflammation. Conversely, sustained *CST7* expression could alter immune cell function in ways that promote persistent inflammation or impair tissue repair. Resolving these possibilities will also be essential for determining whether *CST7* has any translational potential. At present, the context-dependent effects observed across experimental models make it difficult to predict whether increasing or decreasing cystatin F activity would be beneficial. Establishing the relevant cellular source, disease stage, and tissue compartment will therefore be necessary before *CST7* can be meaningfully considered as a therapeutic target.

Ultimately, the evidence reviewed here supports a reevaluation of how cystatin F is viewed within the field. Its remarkable reproducibility across neurodegenerative and neuroinflammatory conditions has firmly established *CST7* as a biomarker of activated neuroimmune cells. However, its conserved biochemical function, immune-enriched expression, and repeated association with lysosomal remodeling collectively provide a strong rationale for testing whether it functions as more than a transcriptional marker. Although definitive mechanistic evidence is still lacking, the convergence of biochemical, transcriptomic, and emerging functional observations provides a compelling rationale to investigate *CST7* as a regulator of neuroimmune responses rather than solely a marker of immune activation. Resolving this question will not only clarify the biological role of cystatin F but may also identify new opportunities to therapeutically modulate lysosomal function and immune activation across a broad spectrum of CNS diseases.

## 8 Conclusion

Over the past decade, advances in transcriptomic and spatial profiling have reshaped our understanding of neuroimmune biology, revealing that many genes once regarded simply as markers of cellular activation may instead participate directly in regulating immune function. Among these emerging candidates, *CST7* represents one of the clearest examples of this transition.

Beyond its value as a transcriptional marker, cystatin F possesses a well-defined biochemical function that directly links it to lysosomal protease regulation, antigen processing, and immune homeostasis. While definitive evidence for these functions in the CNS remains limited, the convergence of molecular biology, immunology, and disease-associated transcriptomic studies suggests that *CST7* is well positioned to influence neuroimmune responses in ways that are only beginning to be understood.

The next phase of neuroimmunology will depend not only on identifying disease-associated cell states, but on defining the molecular mechanisms that establish and maintain them. Whether it ultimately proves to be a biomarker of activation, a regulator of lysosomal homeostasis, or both, understanding the biology of cystatin F promises to provide new insight into the pathways that govern neuroimmune function during health and disease.
